# Emerging Role of Sodium-Glucose Cotransporter-2 Inhibitors in Aortic Stenosis

**DOI:** 10.1016/j.jacbts.2026.101595

**Published:** 2026-06-12

**Authors:** Piotr Mazur, Magdalena Kopytek, Joanna Natorska

**Affiliations:** a University Department of Cardiac Surgery, Leipzig Heart Center, Leipzig, Germany; b Institute of Cardiology, Jagiellonian University Medical College, Kraków, Poland; c John Paul II Hospital, Kraków, Poland

**Keywords:** aortic stenosis, dapagliflozin, flozin, heart disease, SGLT2 inhibitors, empagliflozin

## Abstract

Sodium-glucose cotransporter 2 (SGLT2) inhibitors have emerged as a key drug class in the management of heart failure, expanding far beyond their original use in type 2 diabetes mellitus. Aortic stenosis (AS), the most common valvular heart disease in the aging population, is characterized by progressive valve calcification and chronic left ventricular pressure overload. No disease-modifying pharmacologic treatment has yet been found to slow AS progression or to prevent myocardial remodeling in AS. The authors review the mechanistic rationale for SGLT2 inhibition in the pressure-overloaded heart and summarize the emerging evidence in AS. The authors propose that SGLT2 inhibition may represent a promising strategy targeting both myocardial remodeling and valvular disease biology in AS, warranting further investigation in dedicated trials.

## Highlights

• Cardio- and renoprotective effects expand SGLT2 inhibitor use beyond diabetes.
• This review highlights SGLT2 inhibitors in aortic stenosis and pressure overload.
• Disease-modifying potential of SGLT2 inhibitors in aortic stenosis warrants study.

Aortic stenosis (AS) is the most prevalent valvular heart disease in the Western world, and its incidence continues to rise with population aging. The disease is characterized by progressive fibrocalcific remodeling of the aortic valve (AV) and chronic pressure overload of the left ventricle (LV), resulting from outflow obstruction. Untreated severe AS carries a poor prognosis; although surgical and transcatheter AV replacement (AVR) have transformed the management in the advanced disease, no pharmacologic therapy has yet been found to slow the disease progression or to prevent myocardial remodeling.^1^^,^^2^

Beyond the valve itself, AS affects the myocardial structure and function by implying a chronic pressure overload on the LV. This triggers a cascade of adaptive and maladaptive responses, including cardiomyocyte hypertrophy, interstitial fibrosis, microvascular dysfunction and metabolic reprogramming; these structural and metabolic changes may persist even after valve has been successfully replaced and are increasingly recognized as key determinants of long-term outcomes.^3^^,^^4^

Sodium-glucose cotransporter 2 inhibitors (SGLT2i), originally developed as glucose-lowering agents for type 2 diabetes, have demonstrated robust cardiovascular and renal benefits in large randomized trials studying diverse patient populations.^5^, ^6^, ^7^, ^8^, ^9^ Their clinical efficacy in heart failure (HF) across the spectrum of ejection fraction suggests that these agents exert various effects on the heart that extend beyond glycemic control. Proposed mechanisms include improved myocardial energetics, modulation of inflammatory pathways, attenuation of fibrosis, and improved cellular ionic homeostasis.^10^, ^11^, ^12^

Recent experimental data indicate a possible role of SGLT2i in modulating cardiac remodeling and calcification processes in AS.^13^^,^^14^ These observations raise an intriguing hypothesis: sodium-glucose cotransporter 2 (SGLT2) inhibition may beneficially influence both the pressure-overloaded myocardium and the pathobiology of the stenotic AV. In this review, we discuss the mechanistic rationale for SGLT2 inhibition in AS, summarize emerging experimental and clinical evidence, and highlight key knowledge gaps to guide future research.

## Biology of the Pressure-Overloaded Heart

The defining hemodynamic end effect of AS is the obstruction of blood flow from the LV to the aorta because of fibrocalcific thickening of AV cusps, leading to chronic LV pressure overload. In response to this afterload and increased myocardial wall tension to maintain cardiac output, concentric hypertrophy develops. Although this adaptive remodeling may temporarily preserve systolic function, persistent hemodynamic stress eventually leads to maladaptive structural and functional changes, eventually resulting in impaired subendocardial coronary perfusion, increased myocardial oxygen demand, fibrosis, and finally decompensation.^3^ These changes involve a complex interplay of cardiomyocyte hypertrophy, extracellular matrix expansion, microvascular dysfunction, and metabolic alterations. Collectively, they contribute to progressive diastolic dysfunction, reduced myocardial reserve, and ultimately HF. Importantly, the myocardial phenotype observed in AS shares several molecular features with pressure-overload cardiomyopathy and advanced HF.^3^^,^^4^^,^^15^^,^^16^

### Hypertrophic remodeling

Interstitial and replacement fibrosis represent the pivotal features of myocardial remodeling in AS. Persistent hemodynamic strain and neurohormonal stimulation observed in AS activate a series of profibrotic signaling cascades, such as transforming growth factor-β (TGF-β), galectin-3, and matrix metalloproteinases, which culminate in fibroblast activation, deposition of collagen, and expansion of the extracellular matrix.^4^^,^^15^^,^^16^ Extracellular matrix expansion leads to increased wall stiffness, decreased compliance, poorer diastolic relaxation, and diminished coronary reserve.^17^ Importantly, the extent of myocardial fibrosis has been associated with adverse outcomes after AVR and contributes to persistent postprocedural HF risk, highlighting myocardial remodeling as a key therapeutic target in AS.^18^

### Metabolic reprogramming

Pressure overload also induces substantial metabolic changes within the myocardium. In the later AS stages, myocardial energetic economy is compromised, as evidenced by mitochondrial dysfunction and a metabolic shift from fatty-acid oxidation to glucose use, leading to contractile inefficiency.^19^, ^20^, ^21^ Experimental studies in models of cardiac stress and pressure overload demonstrate that mitochondrial dysfunction is closely linked to altered posttranslational modifications of metabolic enzymes and contractile proteins, including O-GlcNAcylation and other acylation pathways, which impair oxidative phosphorylation and contractile performance.^22^ In parallel, experimental data indicate that increased mitochondrial reactive oxygen species (ROS) production, particularly driven by NOX4 signaling, promotes a shift toward glycolytic metabolism and a proinflammatory phenotype, with reduced oxidative capacity and impaired cellular energetics.^23^ These alterations collectively support the concept that mitochondrial dysfunction and metabolic reprogramming are central features of the pressure-overloaded myocardium.^22^^,^^23^ Down-regulation of fatty-acid oxidation pathways, reduced expression of peroxisome proliferator-activated receptor α, and increased reliance on glucose metabolism have all been reported in the pressure-overloaded heart.^24^ Such metabolic adaptations may initially support energy production, but eventually lead to impaired mitochondrial efficiency and reduced energetic reserve, resembling the metabolic phenotype observed in HF.^25^

### Microvascular dysfunction

In addition to structural remodeling, AS is associated with impaired coronary microvascular function. Reduced capillary density, endothelial dysfunction, and increased myocardial oxygen demand contribute to impaired myocardial perfusion.^17^ Stress cardiac magnetic resonance tomographic studies showed reduced myocardial perfusion reserve in patients with AS, with lower myocardial perfusion reserve associated with subsequent symptom onset and adverse outcomes, including HF and cardiovascular death.^26^ Microvascular dysfunction further exacerbates myocardial ischemia and fibrosis, particularly within the subendocardial layers where wall stress is greatest, and hence represents another potential therapeutic target in the pressure-overloaded heart.

## SGLT2i in the Pressure-Overloaded Myocardium

Although SGLT2i were originally developed to target renal glucose reabsorption, experimental and clinical data suggest that their cardiovascular benefits arise from multiple systemic and cellular mechanisms.^10^, ^11^, ^12^ Several of these pathways are directly relevant to the pathophysiology of pressure-overload cardiomyopathy and may therefore be particularly important in AS.

### Hemodynamic unloading

SGLT2i exert multiple mechanisms relevant for hemodynamic unloading, such as promoting natriuresis and osmotic diuresis through inhibition of glucose and sodium reabsorption in the proximal renal tubule (Central Illustration). These effects reduce volume of plasma and interstitial fluid, leading to decreases in ventricular preload and afterload,^27^ without activating the renin-angiotensin-aldosterone system.^28^ Mild reductions in blood pressure and body weight further decrease the hemodynamic stress on the myocardium.^29^^,^^30^ Such unloading effects may be relevant in the pressure-overloaded heart, where small reductions in afterload can translate into meaningful reductions in myocardial wall stress.

**Central Illustration:**
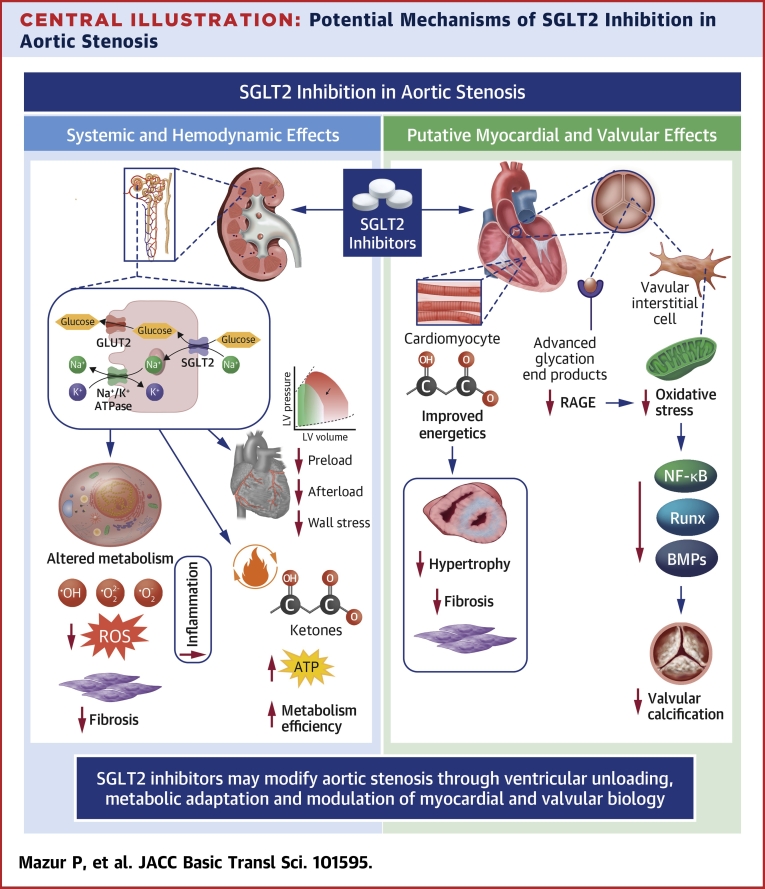
Potential Mechanisms of SGLT2 Inhibition in Aortic Stenosis Sodium-glucose cotransporter 2 inhibitors (SGLT2i) exert systemic metabolic and hemodynamic effects primarily through renal sodium and glucose excretion, resulting in reduced preload and afterload and improved metabolic substrate use. These systemic effects may influence the pressure-overloaded myocardium by improving mitochondrial energetics, modulating intracellular sodium and calcium homeostasis through mechanisms consistent with sodium-hydrogen exchanger inhibition, and attenuating oxidative stress and fibrotic remodeling. In parallel, emerging experimental evidence suggests that SGLT2i may affect pathways involved in aortic valve disease biology, including oxidative stress, inflammatory signaling, and osteogenic differentiation of valvular interstitial cells (VICs). Together, these mechanisms may contribute to improved myocardial recovery after valve replacement and potentially influence the progression of aortic stenosis. AGE = advanced glycation end product; ATP = adenosine triphosphate; ATPase = adenosine triphosphatase; BMP = bone morphogenetic protein; GLUT2 = glucose transporter 2; LV = left ventricular; NF-κB = nuclear factor κB; RAGE = receptor for advanced glycation end products; ROS = reactive oxygen species; Runt = Runt-related transcription factor.

### Metabolic alterations and anti-inflammatory signaling

A growing body of evidence suggests that SGLT2i beneficially influence myocardial energy metabolism. In the myocardium, SGLT2i promote a substrate shift toward oxygen-efficient ketone bodies, improving adenosine triphosphate (ATP) generation and contractility.^31^ Verma et al^31^ demonstrated in a translational study that empagliflozin increases myocardial ATP production, as assessed by phosphorus-31 magnetic resonance spectroscopy, with a corresponding increase in the phosphocreatine/ATP ratio, alongside enhanced myocardial ketone body uptake. These changes were accompanied by improved cardiac work efficiency, reflected by a greater ratio of external cardiac work to myocardial oxygen consumption.^31^ Experimental studies in animal models have shown increased myocardial β-hydroxybutyrate oxidation and improved cardiac output following empagliflozin administration.^32^^,^^33^ In a pressure-overload model, Byrne et al^34^ showed that empagliflozin preserves systolic function in nondiabetic mice subjected to transverse aortic constriction (TAC), as assessed on serial echocardiography, with effects confirmed ex vivo in isolated working heart preparations, demonstrating improved cardiac output and work under controlled preload, afterload, and substrate conditions, independent of circulating factors or ketone availability. Further evidence highlights SGLT2i modulation of cardiac inflammatory signaling pathways.^35^ Yang et al^35^ demonstrated in cellular and animal models that dapagliflozin suppresses NLRP3 inflammasome activation and downstream IL-1β and IL-18 release using western blot and immunohistochemical analyses, accompanied by attenuation of myocardial inflammatory signaling and fibrosis through inhibition of TGF-β/Smad pathways. In animal models, dapagliflozin down-regulates TGF-β and nuclear factor kappa B (NF-κB) signaling, suppressing fibroblast activation and matrix deposition.^36^ In a nondiabetic murine TAC model, empagliflozin improved mitochondrial phosphorylation, reduced H_2_O_2_ production, enhanced mitochondrial biogenesis (PGC-1α/NRF-1/TFAM), and decreased fibrosis on Masson staining, with direct ex vivo confirmation of increased mitochondrial function in isolated LV fibers.^37^ SGLT2i also enhance endothelial function by reducing oxidative stress and increasing nitric oxide bioavailability, leading to lower arterial stiffness and improved flow-mediated dilation.^38^, ^39^, ^40^, ^41^ It has been shown that empagliflozin also limits vascular smooth muscle proliferation and calcification, suggesting antiatherosclerotic and anticalcific potential.^42^

Another proposed important mechanism of SGLT2i cardioprotection involves modulation of intracellular sodium and calcium homeostasis. Experimental studies have suggested that SGLT2i may inhibit the cardiac sodium-hydrogen exchanger 1 (NHE1), leading to reduced intracellular sodium levels and improved calcium handling.^43^ Alsereidi et al^43^ showed that SGLT2i reduce intracellular Na^+^ and secondary Ca^2+^ overload in cardiomyocytes, as assessed by fluorescent ion imaging and electrophysiological measurements, with consequent improvement in calcium cycling and attenuation of cellular stress signaling (including CaMKII and calcineurin-NFAT pathways, as well as ROS-dependent MAPK signaling). In the light of available evidence, the involvement of NHE1 inhibition remains putative though, and these effects are likely indirect rather than reflecting direct target engagement.

**Figure 1:**
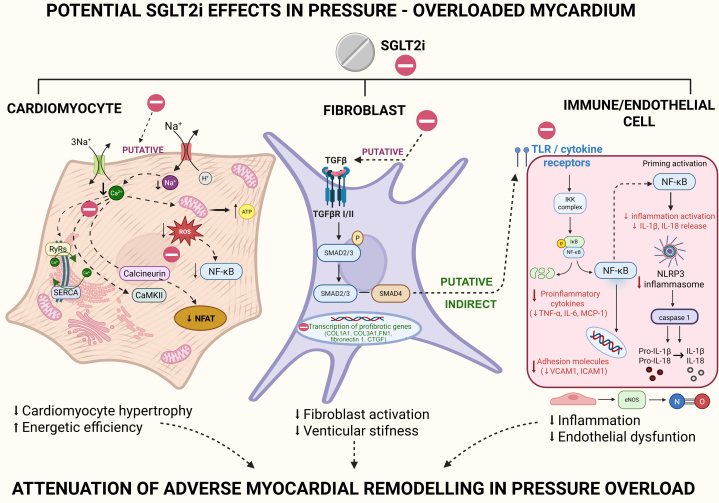
SGLT2 Inhibitory Effects in the Pressure-Overloaded Myocardium Schematic representation of the proposed sodium-glucose cotransporter 2 inhibitor (SGLT2i) mechanisms modulating myocardial remodeling in aortic stenosis, integrating effects across cardiomyocytes, cardiac fibroblasts, and endothelial and immune cells. In cardiomyocytes, SGLT2i are associated with improved energetics (increased adenosine triphosphate [ATP] production, reduced mitochondrial reactive oxygen species [ROS]) and may indirectly modulate intracellular Na^+^/Ca^2+^ handling, possibly via inhibition of the sodium-hydrogen exchanger, thereby attenuating CaMKII, calcineurin-NFAT, and nuclear factor κB (NF-κB) signaling pathways linked to hypertrophy and inflammation. In cardiac fibroblasts, profibrotic signaling is driven by transforming growth factor-β (TGF-β) binding to its receptor complex (TGFβR I/II), activating SMAD2/3-SMAD4-dependent transcription of extracellular matrix genes. SGLT2i are proposed to attenuate fibroblast activation and matrix deposition; however, these effects are likely indirect and not due to direct inhibition of TGF-β signaling. In endothelial and/or immune cells, inflammatory activation involves NF-κB-dependent priming and subsequent NLRP3 inflammasome activation, leading to caspase-1-mediated maturation of interleukin (IL)–1β and IL-18. SGLT2i are associated with reduced inflammatory signaling, decreased cytokine release, and improved endothelial function, including enhanced nitric oxide bioavailability. Collectively, these predominantly indirect and multicellular effects are associated with attenuation of adverse myocardial remodeling, reduced fibrosis, and improved ventricular function in pressure overload, while direct cellular targets of SGLT2i within the myocardium remain uncertain. COL1A1 = collagen type I α1 chain; COL3A1 = collagen type III α1 chain; CTGF = connective tissue growth factor; FN1 = Fibronectin-1; ICAM1 = intercellular adhesion molecule 1; MCP-1 = monocyte chemotactic protein-1; NFAT = nuclear factor of activated T-cells; RyRs = ryanodine receptors; SERCA = sarcoplasmic/endoplasmic reticulum Ca2+-ATPase; TLR = Toll-like receptor; TNF-α = tumor necrosis factor-α; VCAM1 = vascular cell adhesion molecule 1.

Collectively, SGLT2i exert anti-inflammatory, antioxidant, antifibrotic, and metabolic effects (Figure 1). Improvements in mitochondrial function, oxidative stress, and cellular ionic balance may all contribute to reduced inflammatory signaling in the myocardium.

### Direct vs indirect mechanisms of SGLT2i

A key unresolved question regarding the cardiovascular effects of SGLT2i is whether they result from direct inhibition of myocardial or valvular SGLT2 or rather indirect systemic mechanisms. SGLT2 is expressed predominantly in the renal proximal tubule, where it mediates sodium and glucose reabsorption. Evidence for SGLT2 expression in the myocardium remains limited and somewhat controversial. Transcriptomic studies have reported SGLT2 expression in failing human myocardium and in patients with severe AS.^14^^,^^44^ However, bulk transcriptomic approaches cannot reliably determine the cellular origin of detected transcripts. Signals attributed to cardiomyocytes may instead reflect endothelial cells, inflammatory infiltrates, or disease-related changes in tissue composition. Consequently, many of the proposed “direct” molecular effects of SGLT2i may represent indirect downstream consequences of metabolic, hemodynamic, or ionic changes induced by these drugs. Distinguishing between direct target engagement and secondary cellular responses remains an important priority for future mechanistic studies, integrating cell-specific transcriptomics, spatial biology, and pharmacologic target engagement.

## AS Biology

**Figure 2:**
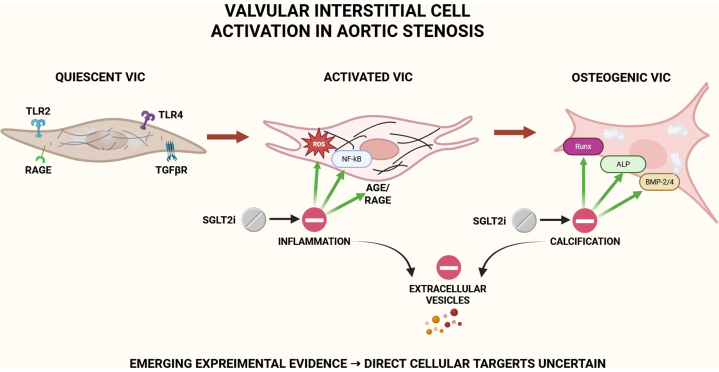
Valvular Interstitial Cell Activation and Potential Modulation by SGLT2i in Aortic Stenosis Schematic overview of valvular interstitial cell (VIC) activation and osteogenic differentiation in calcific aortic stenosis, with potential points of modulation by SGLT2i. Quiescent VICs transition to an activated phenotype in response to inflammatory and metabolic stimuli, including Toll-like receptor (TLR) signaling, receptor for advanced glycation end products (RAGE) activation, and TGF-β pathways. This process is associated with increased oxidative stress and activation of nuclear factor κB (NF-κB), promoting inflammatory signaling and extracellular matrix remodeling. Activated VICs subsequently undergo osteogenic differentiation characterized by up-regulation of Runt-related transcription factor 2 (Runx2), bone morphogenetic proteins (BMP-2/4), and alkaline phosphatase (ALP), leading to progressive valvular calcification. Emerging experimental evidence suggests that SGLT2i may attenuate these processes by reducing oxidative stress, inflammation, and extracellular vesicle–mediated signaling; however, direct cellular targets within the valve remain uncertain. Abbreviations as in Figure 1.

Calcific AS is recognized as an active biological process rather than a purely degenerative condition. The disease evolves through sequential stages involving lipid infiltration, inflammation, fibrocalcific remodeling, and osteogenic differentiation of valvular interstitial cells (VICs) (Figure 2).^2^ The formation of foam cells (transformed monocytes and macrophages) results from dysregulation of modified lipoproteins influx and efflux. VICs experience inflammatory activation, modulated by the overexpression of NADPH-oxidase 2, the principal cellular source of ROS. The augmented generation of ROS activates the Smad1/5/8 and Wnt/β-catenin signaling pathways, leading to overexpression of the master osteoblast transcription factor, Runt-related transcription factor 2/core-binding factor α-1, and the synthesis of calcification-associated proteins, such as bone morphogenetic proteins (BMPs), osteopontin, and osteoprotegerin.^2^^,^^45^ BMP-2 and BMP-4 facilitate the secretion of osteopontin by up-regulating alkaline phosphatase. Consequently, this leads to the remodeling of the extracellular matrix and the deposition of osteoblast-like cells.^2^ Notably, osteopontin also serves as a significant proinflammatory cytokine that is instrumental in the recruitment of leukocytes, and its levels have been correlated with an elevated cardiovascular risk among patients with diabetes.^46^ The process of valvular ossification and calcification is also regulated by NF-κB expression.^47^ NF-κB serves as a master regulator of inflammatory responses, playing a central role in both the progression and resolution phases of inflammation. Overexpression of NF-κB has been associated with the development and progression of atherosclerosis.^47^ The NF-κB subunit p65/c-Rel, which is activated by TNF-α, critically regulates not only inflammation, but also the expression of tissue factor, initiating the extrinsic pathway of blood coagulation.^47^ A significant expression of tissue factor has been reported in stenotic AV, in association with valvular fibrin deposition and an increased number of macrophages.^48^^,^^49^ It has been postulated that diabetes may initiate or exacerbate valvular calcification through complex interactions of vascular and inflammatory cells.^50^ AVs from patients with diabetes exhibit significant presence of C-reactive protein–positive areas, which correlates with heightened coagulation activation.^51^ It has also been reported that advanced glycation end products (AGEs) accumulate in the stenotic AV, potentially being a contributor to the progression of AS.^52^^,^^53^ AGEs, a complex and heterogeneous group of compounds, are rapidly generated following exposure to elevated blood glucose levels, and are implicated in diabetes-related complications, altering the tissue structure and/or function by cross-linking of intra- and extracellular matrix proteins.^54^ Moreover, AGEs, when they bind to their cell surface receptor (RAGE), affect multiple cellular processes, including ROS generation, inflammation, coagulation, and fibrinolysis.^50^

In vitro investigations using VICs have demonstrated that the inhibition of either ROS or NF-κB effectively prevents cellular calcification.^55^ Animal models of AS have revealed that the accumulation of AGEs within AV activates the AGEs/RAGE axis–mediated NF-κB overexpression, leading to the osteoblastic differentiation of VICs.^54^^,^^56^ In diabetic patients with AS undergoing surgical AVR, the accumulation of valvular AGEs was found to be 6 times higher, and plasma levels of AGEs were 12 times greater, in comparison with matched nondiabetic patients.^52^ Similarly, both valvular and plasma RAGE levels were elevated in diabetic AS patients relative to their nondiabetic counterparts.^52^ Increased expression of AGEs/RAGE was accompanied by enhanced valvular expression of NF-κB, coagulation factors and calcification marker, BMP-2.^55^ Given the central roles of inflammation, oxidative stress, and metabolic dysfunction in valvular degeneration, these pathways may represent potential therapeutic targets in AS.

## Preclinical Evidence

Experimental studies have begun to explore the potential role of SGLT2i in models of pressure-overload cardiomyopathy mimicking key features of myocardial remodeling observed in AS. These models typically involve TAC or supravalvular banding that reproduce chronic outflow obstruction. In murine TAC models, empagliflozin has been shown to attenuate ventricular hypertrophy, improve myocardial function, and reduce arrhythmogenic remodeling.^34^^,^^57^ Importantly, these effects were observed even in nondiabetic animals, suggesting that the cardioprotective actions of SGLT2 inhibition extend beyond glucose lowering. Several pathways appear to contribute to these effects. Experimental data suggest that SGLT2i may influence intracellular ionic homeostasis, potentially through inhibition of the cardiac NHE1, resulting in reduced intracellular sodium accumulation and improved calcium handling within cardiomyocytes. In a murine TAC model of pressure overload, SGLT2 inhibition with empagliflozin reduced intracellular Na^+^ accumulation consistent with NHE1 modulation, although direct inhibition of the exchanger was not demonstrated.^58^ Nevertheless, this shift in ionic balance may improve mitochondrial function, reduce oxidative stress, and limit the activation of hypertrophic and fibrotic signaling cascades. In other TAC models, improved calcium cycling and reduced arrhythmogenic activity have been reported following empagliflozin treatment, suggesting potential antiarrhythmic effects in the pressure-overloaded heart.^57^ A preliminary study with a rat model of AS induced by supravalvular aortic banding demonstrated that 8-week empagliflozin treatment attenuated adverse ventricular remodeling, as evidenced by reduced LV dilatation and mass on echocardiography, and decreased interstitial collagen fraction on histologic analysis, while improving diastolic function reflected by prolongation of isovolumetric relaxation time, compared with untreated animals.^59^ In this study, animals treated with empagliflozin showed reduced myocardial oxidative stress, lower levels of interstitial collagen deposition and decreased activation of profibrotic signaling pathways compared with untreated controls.

In addition to myocardial effects, emerging experimental evidence suggests that SGLT2 inhibition may influence valvular biology. In 2024, Valerio et al^60^ performed RNA sequencing of 39 stenotic AVs, investigating sirtuin-1 expression, as a potential regulator of AS-related processes. VICs with sirtuin-1 gene knockdown exhibited increased calcium deposition, but exposure to medium with dapagliflozin reduced calcification and increased NO levels.^60^ Hmadeh et al^61^ studied valves from 105 patients undergoing surgical AVR, including 90 with severe AS, focusing on extracellular vesicles trapped within the native AV. They demonstrated that calcified valves constitute a major reservoir of procoagulant cell-derived extracellular vesicles, which trigger up-regulation of different proteins, including SGLT2 in valvular endothelial cells. Calcified valves have been shown to contain large numbers of procoagulant extracellular vesicles capable of promoting endothelial dysfunction and inflammatory activation. Ex vivo experiments showed that increased SGLT2 levels induce endothelial valvular dysfunction, recruitment of inflammatory cells and thrombogenicity, while treatment with empagliflozin abolished these effects, which was likely attributable to the high effectiveness of empagliflozin to impede the sustained pro-oxidant activator signal and NF-κB activation.^61^

These preclinical findings suggest that potential beneficial mechanisms of SGLT2i action may include attenuation of cardiomyocyte hypertrophy, reduction of myocardial fibrosis, improvement of mitochondrial energetics, modulation of intracellular ionic homeostasis, and potential effects on inflammatory and oxidative pathways involved in valvular degeneration. Despite these promising observations, important limitations should be acknowledged, as many experimental models represent relatively acute forms of pressure overload and may not reproduce the slow progression of calcific AS in humans. In addition, the extent to which these effects reflect direct actions of SGLT2i within myocardial or valvular tissue, as opposed to systemic metabolic and hemodynamic effects, remains unclear.

## Current Clinical Evidence in AS

Although the cardiovascular benefits of SGLT2i are now well established in HF or CKD, the clinical evidence regarding their role in AS remains sparse. Nevertheless, an increasing number of observational studies, registry analyses, and early randomized trials have begun to explore the potential role of SGLT2 inhibition on disease progression, myocardial recovery, and clinical outcomes in this population. The available data currently fall into 3 main categories: observational studies evaluating the association between SGLT2i exposure and AS progression, registry analyses examining outcomes after AVR, predominantly transcatheter AVR (TAVR), and randomized clinical trials investigating the effects of SGLT2i in patients undergoing TAVR.

### Observational studies in AS

One of the largest studies addressing the relationship between SGLT2i use and the natural history of AS was a multicenter observational retrospective analysis conducted by Shah and colleagues in 2025, which included 11,698 patients with early AS stages, ranging from aortic sclerosis to moderate AS, who underwent serial echocardiographic evaluation over a 3-year follow-up period.^13^ Among these individuals, 448 patients were treated with SGLT2i for diabetes management. After adjustment for baseline characteristics and comorbidities, SGLT2i therapy was associated with a significantly lower risk for progression to severe AS (HR: 0.61) compared with patients not receiving SGLT2i.^13^ In addition to the reduced risk for developing severe disease, the rate of echocardiographic progression (assessed by changes transvalvular gradients and valve area over time) was slower in the SGLT2i–treated cohort. Sensitivity analyses suggested that the observed associations remained consistent across different subgroups, including patients with different renal function, glycemic control, and baseline AS severity. The beneficial association appeared also more pronounced in patients with longer cumulative exposure to SGLT2i, suggesting a possible duration-dependent effect.^13^ These findings point to the possibility that SGLT2 inhibition may influence the biological processes driving valvular degeneration in AS, but the retrospective nature of the evidence precludes causal inference and warrants randomized studies on the topic.

### SGLT2i and outcomes after AVR

Although AVR effectively relieves the AS-related mechanical outflow obstruction, many patients continue to experience persistent myocardial dysfunction and HF related events postinterventionally.

Several observational studies and registry analyses have evaluated the outcomes among SGLT2i–treated patients following TAVR. In a multicenter international registry including 311 diabetic patients with severe AS undergoing TAVR, Paolisso et al^62^ examined the association between SGLT2i therapy and postprocedural outcomes. Approximately one-quarter of patients in this cohort received SGLT2i for diabetes management. Despite lower baseline LV ejection fractions, patients treated with SGLT2i demonstrated greater recovery of ventricular function during follow-up compared with those not receiving SGLT2i.^62^ In addition, SGLT2i therapy was associated with lower rates of major adverse cardiovascular events during the follow-up: after 2 years, patients receiving SGLT2i experienced fewer HF hospitalizations and lower rates of all-cause mortality compared with control patients.^62^ In contrast, Yasmin et al,^63^ in a recent retrospective analysis of 21,828 patients with HF undergoing TAVR, of whom 2,464 received SGLT2i, reported that after propensity score matching (2,039 pairs) the primary composite outcome (acute myocardial infarction, stroke, all-cause mortality, and acute HF) was not statistically different at 6 months among patients receiving SGLT2i compared with those receiving standard care. SGLT2i use was associated with a lower risk for acute myocardial infarction and all-cause hospitalization.^63^ However, in that study, exposure to SGLT2i was defined at the index date and treated as fixed, hence treatment duration, potential discontinuation or switching during follow-up was not analyzed.

Another recent retrospective analysis by Fahoury et al^64^ using the TriNetX Research Network included 4,077 TAVR patients receiving SGLT2i, along with 854 patients treated with glucagon-like peptide 1 receptor antagonists and 584 individuals taking both. SGLT2i monotherapy after TAVR in this report decreased the rate of arrhythmias (HR: 0.70), while a combination therapy showed decreased mortality (HR: 0.42), acute HF (HR: 0.49), myocardial infarction (HR: 0.50), and arrhythmia (HR: 0.40).^64^ In another study using the TriNetX Research Network, which included 2,297 pairs of matched patients treated with TAVR with or without subsequent SGLT2i treatment, followed for a median of 1.4 years, Morel et al^65^ reported that SGLT2i use was associated with a significantly lower risk for all-cause mortality (HR: 0.83) and, interestingly, protective against bioprosthetic valve failure (HR: 0.62).

Additional preliminary data have been reported in the EASTER-HF study, a small prospective investigation evaluating the effects of empagliflozin in patients with severe AS and newly diagnosed HF undergoing surgical AVR.^66^ In that study, empagliflozin use was associated with improvements in LV ejection fraction and reductions in HF hospitalizations during the 6-month follow-up period. The limited sample size (n = 40) and nonrandomized design necessitate cautious interpretation of these findings.

### Evidence from randomized controlled trials

Randomized evidence evaluating SGLT2i use in patients with AS has only recently begun to emerge. The DapaTAVI (Dapagliflozin After Transcatheter Aortic Valve Implantation) trial represents the first large randomized study specifically investigating the effects of SGLT2i therapy in patients undergoing TAVR.^67^ In that multicenter randomized controlled trial, 1,222 patients with severe AS undergoing TAVR and presenting with additional risk factors, including HF, chronic kidney disease, diabetes, and LV systolic dysfunction, were randomized to receive dapagliflozin or standard care following TAVR. The primary endpoint was a composite of all-cause mortality or worsening HF during follow-up. At 1 year, treatment with dapagliflozin resulted in a 28% relative risk reduction in the primary composite endpoint, compared with standard therapy alone.^67^ This reduction was driven primarily by a lower incidence of HF hospitalizations among patients receiving dapagliflozin. Importantly, the beneficial effects of dapagliflozin were observed across different subgroups, including patients with preserved LV ejection fractions and older individuals undergoing TAVR. These findings are consistent with prior HF trials demonstrating the efficacy of SGLT2i across a broad spectrum of patients, irrespective of diabetic status.^8^^,^^9^ The safety profile observed in the trial was generally favorable and consistent with previous studies of SGLT2i; the overall adverse event rates remained low and no new safety concerns were identified.^67^ Genital infections occurred 3 times more often in patients receiving dapagliflozin (still, the incidence was 1.8%) and hypotension occurred twice as often, when compared with control subjects (6.6% vs 3.6%, respectively), with no other differences.^67^

### Ongoing trials

An overview of ongoing registered studies evaluating SGLT2 inhibition in AS is presented in Table 1. These investigations address different stages of disease and clinical contexts, including prevention of myocardial remodeling in moderate AS, optimization of cardiac recovery following valve intervention, and long-term cardiovascular risk reduction after TAVR. Collectively, they explore the hypothesis that SGLT2i may modulate both myocardial adaptation to pressure overload and clinical outcomes after valve intervention.

**Table 1:** Active Registered Studies on the Use of SGLT2i in Patients With AS

ClinicalTrials.gov ID	Title	Status	Treated Condition	Intervention	Primary Outcome	Single Center vs Multicenter	Enrollment	Start Date	End Date	Country
NCT05672836	ENAVO-TAVR	Recruiting	AS post-TAVR and HF with LVEF ≥ 40%	RCT: enavogliflozin 0.3 mg qd vs placebo	Composite of MACE or hospitalization for HF at 12 mo after TAVR	Multicenter	1,040	2024	2027	South Korea
NCT06469645	The Role of SGLT2i in Management of Moderate AS	Recruiting	Moderate AS	RCT: empagliflozin 10 mg qd vs placebo	Indexed cardiac extracellular volume on CMR at 6 mo	Single center	104	2024	2026	United Kingdom
NCT06090591	Cardiology Research Dubrava Prospective Registry	Recruiting	Patients implanted with CIED or with VTE, ACS, or TAVR	Observational: SGLT-2i therapy, CIED implantation, thromboaspiration	CV death, freedom from atrial arrhythmias, bleeding complications, NT-proBNP levels at 10 y	Single center	3,000	2023	2033	Croatia
NCT05241431	DAPAS	Unknown (last known status was recruiting)	AS scheduled for TAVR and HF with LVEF ≥ 40%	RCT: dapagliflozin 10 mg qd vs placebo	Composite endpoint of changes in LV mass, systolic function, eGFR, and serum NT-proBNP at 12 mo after TAVR	Single center	106	2022	2024 (?)	Denmark

ACS = acute coronary syndrome; AS = aortic stenosis; CIED = cardiac implantable electronic device; CMR = cardiac magnetic resonance; CV = cardiovascular; DAPAS = Effect of Dapagliflozin on Myocardial and Renal Function Following Aortic Valve Stenosis Intervention; eGFR = estimated glomerular filtration rate; ENAVO-TAVR = Enavogliflozin Outcome Trial in Patients With Severe Aortic Stenosis After Transcatheter Aortic Valve Replacement; HF = heart failure; LV = left ventricular; LVEF = left ventricular ejection fraction; MACE = major adverse cardiovascular events; NT-proBNP, N-terminal pro–B-type natriuretic peptide; RCT = randomized controlled trial; SGLT2i = sodium-glucose linked transporter 2 inhibitors; TAVR = transcatheter aortic valve replacement; VTE = venous thromboembolism.

One ongoing randomized study evaluates empagliflozin in patients with moderate AS (NCT06469645). This trial focuses on myocardial remodeling, with the primary endpoint defined as changes in myocardial extracellular volume fraction assessed by cardiac magnetic resonance. By targeting myocardial fibrosis, this study is intended to determine whether SGLT2i may influence structural myocardial changes during earlier stages of AS progression (Table 1).

The ENAVO-TAVR (Enavogliflozin Outcome Trial in Patients With Severe Aortic Stenosis After Transcatheter Aortic Valve Replacement) trial (NCT05672836) is currently evaluating enavogliflozin in patients undergoing TAVR with HF and preserved or mildly reduced LV ejection fractions. The primary endpoint is a composite of major adverse cardiovascular events or HF hospitalization at 12 months (Table 1). Similarly, the DAPAS (Effect of Dapagliflozin on Myocardial and Renal Function Following Aortic Valve Stenosis Intervention) study (NCT05241431) investigates dapagliflozin in patients undergoing TAVR, with endpoints including changes in LV mass, systolic function, renal function, and circulating biomarkers, such as N-terminal pro–B-type natriuretic peptide. By incorporating imaging, functional, and biomarker endpoints, this trial may provide mechanistic insights into the effects of SGLT2 inhibition on myocardial remodeling after relief of pressure overload (Table 1). In addition to randomized trials, prospective registries are also collecting long-term observational data on patients receiving SGLT2i after cardiovascular interventions. The Cardiology Research Dubrava Prospective Registry (NCT06090591) includes patients undergoing TAVR and is intended to evaluate long-term cardiovascular outcomes, arrhythmia burden, and biomarker trajectories over extended follow-up periods (Table 1).

## Translational Perspective

The pathophysiology of AS reflects a complex interplay between progressive valvular degeneration and myocardial (mal)adaptation to chronic pressure overload. Although AVR effectively relieves the mechanical obstruction, myocardial remodeling often persists and continues to influence the clinical outcomes. An increasing body of evidence suggests that metabolic dysfunction, oxidative stress, inflammation, and fibrosis represent shared biological pathways linking pressure-overload cardiomyopathy and valvular degeneration. SGLT2i have been shown to influence many of these pathways through mechanisms involving improved myocardial energetics, modulation of inflammatory signaling, and attenuation of fibrotic remodeling.^10^, ^11^, ^12^ SGLT2i may target the pressure-overloaded myocardium, while potentially influencing biological processes involved in valvular calcification as well. Although current data remain limited, the convergence of mechanistic insights, experimental evidence, and early clinical observations provides a strong rationale for further research, both mechanistic and clinical.

## Future Directions

Several key questions remain regarding the potential role of SGLT2i in AS. First, prospective randomized trials are required to determine whether SGLT2i can modify the progression of early or moderate AS. Such studies would ideally incorporate serial imaging of valvular calcification and myocardial fibrosis.

Second, additional research is needed to clarify the role of SGLT2i in promoting myocardial recovery after AVR, including both surgical and TAVR patients, as well as patients with bicuspid and tricuspid AV.

Third, mechanistic studies are urgently needed to explore whether SGLT2i exert direct effects on VICs and/or cardiomyocytes.

Finally, future research may also investigate whether SGLT2i could influence the durability of bioprosthetic valves or modulate structural valve degeneration. Together, these research directions could help define the potential role of SGLT2i in a broader pharmacologic strategy aimed at modifying both myocardial remodeling and disease progression in the context of lifetime management of AS.

## Conclusions

Increasing evidence indicates that several mechanisms associated with SGLT2 inhibition, including hemodynamic unloading, improved myocardial energetics, modulation of inflammatory signaling and attenuation of fibrotic remodeling, are relevant to the pathophysiology and pathobiology of AS. Preclinical studies demonstrate beneficial effects on myocardial hypertrophy, oxidative stress, and fibrosis in pressure-overload models, while emerging clinical data suggest improved ventricular recovery and reduced HF events in patients undergoing valve intervention. Although current evidence remains limited, the convergence of mechanistic insights, experimental observations, and early clinical studies provides a strong rationale for further investigation. Ongoing trials will clarify whether SGLT2i can improve myocardial adaptation to pressure overload and potentially contribute to disease-modifying strategies in AS.

Perspectives **COMPETENCY IN MEDICAL KNOWLEDGE:** SGLT2i may influence multiple biological pathways relevant to aortic stenosis, including myocardial remodeling, fibrosis, inflammation, and cellular energetics.

**COMPETENCY IN PATIENT CARE:** Emerging clinical evidence suggests that SGLT2i may improve outcomes after aortic valve interventions, particularly by reducing HF events.

**TRANSLATIONAL OUTLOOK:** Prospective randomized trials are needed to determine whether SGLT2i can modify myocardial remodeling and alter the natural history of aortic stenosis.

## Funding Support and Author Disclosures

This work was supported by the grant from the Polish National Science Centre (UMO-2023/51/B/NZ5/00268 to Dr Natorska). The authors have reported that they have no relationships relevant to the contents of this paper to disclose.
